# Poisoning Regulation, Research, Health, and the Environment: The Glyphosate-Based Herbicides Case in Canada

**DOI:** 10.3390/toxics11020121

**Published:** 2023-01-26

**Authors:** Marie-Hélène Bacon, Louise Vandelac, Marc-André Gagnon, Lise Parent

**Affiliations:** 1 Ecohealth Research Collective on Pesticides, Policies and Alternatives (CREPPA), Institute of Environmental Sciences, Université du Québec à Montréal (UQAM), Montréal, QC H3C 3P8, Canada; 2 Ecohealth Research Collective on Pesticides, Policies and Alternatives (CREPPA), Department of Sociology and Institute of Environmental Sciences, Université du Québec à Montréal (UQAM), Montréal, QC H3C 3P8, Canada; 3 School of Public Policy and Administration, Carleton University, Ottawa, ON K1S 5B6, Canada; 4 Ecohealth Research Collective on Pesticides, Policies and Alternatives (CREPPA), Science and Technology Department, Université TÉLUQ, Montréal, QC H2S 3L5, Canada

**Keywords:** pesticide, glyphosate, glyphosate-based-herbicides, herbicide, Canada, Quebec, Health Canada, regulation, scientific evaluation, Monsanto, regulatory capture, environmental health

## Abstract

Despite discourse advocating pesticide reduction, there has been an exponential increase in pesticide use worldwide in the agricultural sector over the last 30 years. Glyphosate-Based Herbicides (GBHs) are the most widely used pesticides on the planet as well as in Canada, where a total of almost 470 million kilograms of declared “active” ingredient glyphosate was sold between 2007 and 2018. GBHs accounted for 58% of pesticides used in the agriculture sector in Canada in 2017. While the independent scientific literature on the harmful health and environmental impacts of pesticides such as GBHs is overwhelming, Canada has only banned 32 “active” pesticide ingredients out of 531 banned in 168 countries, and reapproved GBHs in 2017 until 2032. This article, based on interdisciplinary and intersectoral research, will analyze how as a result of the scientific and regulatory captures of relevant Canadian agencies by the pesticide industry, the Canadian regulation and scientific assessment of pesticides are deficient and lagging behind other countries, using the GBH case as a basis for analysis. It will show how, by embracing industry narratives and biased evidence, by being receptive to industry demands, and by opaque decision making and lack of transparency, Health Canada’s Pest Management Regulatory Agency (PMRA) promotes commercial interests over the imperatives of public health and environmental protection.

## 1. Introduction

With one-third of the world’s land and almost 75% of the world’s freshwater resources devoted to crop or livestock production [1], the global food system, which relies heavily on intensive industrial agriculture and its chemical products, is one of the main drivers of planetary boundary (PB) crossings, causing the world to reach dangerous environmental tipping points. The six PB already transgressed includes the two fundamental ones, climate change and biosphere integrity, as well as biogeochemical flows, land system change, freshwater change (green water) and novel entities [2,3,4]. From genetically modified organisms (GMOs) to chemical substances, plastics and nanoproducts, a large amount of these novel entities is being introduced into the environment, frequently for agriculture purposes. This has heavy negative impacts on biodiversity and environmental degradation worldwide.

Despite discourses advocating pesticide reduction, their sales, toxicity and use in agriculture have been growing exponentially worldwide, with a 58% sales increase over the last 30 years (1990–2020) [5]. In 20 years, the value of the international market for agricultural pesticides has almost doubled, going from EUR 30 billion in 2000 to about EUR 52 billion in 2019, and the world exportation market of pesticides has tripled in value over the same period, increasing from EUR 10 billion in 2000 to EUR 32 billion in 2019 [6]. Pesticides which are banned for use in Europe for health and toxicity reasons are being exported to countries in Africa, South-East Asia or Central and South America, like Brazil [7,8]. This contributes to increasing health and environmental problems, and also raises issues of human rights [9,10]. In 2018 alone, European Union (EU) member countries approved the export of more than 80,000 tons of highly toxic pesticides containing banned substances, thus contributing to the 385 million annual cases of serious and unintentional poisoning, as well as the 11,000 annual pesticide-related deaths worldwide [11,12].

Research carried out in molecular biology, on animal models, in epidemiology, in occupational health studies, and in meta-analyses have all documented and demonstrated the harmful impacts and mechanisms of action of pesticides on human health [13,14,15,16,17,18]. In France, a report produced by the Institut national de la santé et de la recherche médicale (Inserm) on the chronic toxicity of pesticides showed strong evidence of pesticide-linked cognitive disorders, prostate cancer, multiple myeloma, non-Hodgkin’s lymphoma (NHL), hematological malignancies, and congenital malformations and leukemia in children exposed during pregnancy [19]. Agrican, one of the largest cohort studies in the world on cancers in the agricultural workplace, confirmed increased risks such as hematological malignancies and prostate cancer [20]. These toxic chemicals, now found in soil, water, air, food, feed, and the human body, have fundamentally important consequences on public health, ecosystems and biodiversity. The specter of a Silent Spring, predicted 60 years ago in Rachel Carson’s timely book on pesticides, is becoming a reality [21], with nearly 100 million birds killed annually in the United States alone, and nearly three billion fewer birds than in 1970 [22,23].

Glyphosate-based herbicides (GBHs), used on many cultures, but especially on GMOs, designed to absorb them without dying, are the most widely sold herbicides in the world. Agricultural GMOs such as soybeans, corn and canola are not only designed to tolerate GBHs, but the quantity of GBHs used on these GM crops is much higher than in regular crops. In Canada, herbicides accounted for 77% of pesticide sales in agriculture in 2019 [24] and GBHs are by far the most widely used herbicide, with a total of almost 470 million kilograms of active ingredient (kg a.i.) of glyphosate sold between 2007 and 2018 [25]. In comparison, 2,4-D sales, also among the main herbicides used in the country, were 14 times lower [25]. Several studies have shown the impacts of glyphosate and GBHs on health [26,27,28,29,30,31,32,33,34,35,36]. For example, a meta-analysis by Zhang et al. showed an increase of 41% in cancer risk for agricultural workers with the highest exposure to complete GBH formulations [37]. It is difficult to comprehend that while the independent scientific literature on the harmful health and environmental impacts of pesticides such as GBHs is overwhelming, Canada reapproved GBHs in 2017 until 2032. It also had 7593 pesticides approved for use in 2019, and only 32 banned [24,38].

In North America, but globally as well, some factors have contributed more than others to this situation. The United States, Canada, Brazil and Argentina have massively adopted the use of genetically modified corn, soya, and canola since the 1990s, and also expanded GBH use to other cultures, forests, railroad sidetracks, and more [39]. Over the last three decades, an important economic concentration of firms in the agrochemical sector has been taking place, largely through mergers and acquisitions. Of the 16 agrochemical firms in this sector in 1990, there are now only 4 major firms left [6,40]. With 6 firms owning 78% of the world’s agrochemicals market share in 2020, and 2 controlling 40% of patented seeds [40], this industry which now resembles a cartel, has been using “behind-the-scenes” strategies to protect product sales by hiding away and downplaying potential harms, refuting external scientific research and influencing regulators worldwide. These proactive corporate strategies used to shape scientific narratives about pesticides have had a profound impact on their evaluation and regulation, as it has been taken up by both regulatory bodies and the media around the world.

This article, based on the interdisciplinary and intersectoral research of the Collectif de recherche écosanté sur les pesticides, les politiques et les alternatives (CREPPA), will analyze how, as a result of the scientific and regulatory captures of relevant Canadian agencies by the pesticide industry, the Canadian regulation and scientific assessment of pesticides are deficient, using the GBH case as a basis for analysis. Among other things, the regulatory framework focuses solely on the “active” ingredient and does not take into account the full formulation of GBHs, leading to an underestimation of their toxicity and their health and environmental impacts. This article will also assert that the lack of transparency regarding the evaluation and decision-making processes of Health Canada’s Pest Management Regulatory Agency (PMRA), and the lack of public access to even the most basic data such as accurate pesticide sales, combined with an unhealthy intimacy between Canadian regulatory bodies and agrochemical companies undermine the protection of Canadian public health and environment.

## 2. Canadian Pesticide Evaluation and Regulation: Deficient and Lagging behind Other Countries

The Health Canada Pest Management Regulatory Agency (PMRA) is responsible for pesticide regulation in Canada. Acting under the authority of the Pest Control Products Act (PCPA), the PMRA is in charge of evaluating pesticides before their registration, as well as their re-evaluation after 15 years “to ensure they pose minimal risk to human health and the environment” [41]. For many years now, the PMRA has been working closely with its counterpart in the United States, the Environmental Protection Agency (EPA), and has aligned its policies, its pesticide evaluation parameters and methods, and its regulatory orientations with the American ones.

Because the evaluation of pesticides is risk-based in Canada—meaning that the main objective is to “prevent unacceptable risks to individuals and the environment from the use of pest control products” [42]—the precautionary principle is not applied and does not have any legal weight. To determine if a “risk is acceptable”, the PMRA uses manufacturer’s studies and data, and the concept of the “value” of the pesticide which is determined by “the product’s actual or potential contribution to pest management” as well as its “(a) efficacy; (b) effect on host organisms in connection with which it is intended to be used; and (c) health, safety and environmental benefits and social and economic impact.” [42]. It is important to note here that only the ingredient declared “active” in a pesticide by the manufacturer is evaluated and regulated or even taken into account for the sales’s estimation in Canada.

Glyphosate, and not GBHs, is thus the only focus of the regulatory assessment and established norms, for instance in food or water. In its evaluation of GBHs, the PMRA did not consider all co-formulants even though these mixtures of compounds can be highly toxic. They were found to be up to 1000 times more toxic than the so-called “active” ingredient in eight of the nine best-selling pesticides worldwide [43]. Bayer Crop ScienceMonsanto’s Roundup WeatherMax(Bayer CropScience Inc., Dovercourt, Canada), a GBH widely used in the Canadian province of Quebec, contains formulants such as arsenic, chromium, and lead, which are known to be toxic and endocrine disruptors [44], but when assessing environmental and health impacts of pesticides, the PMRA ignores surfactants, heavy metals, petroleum and other “contaminants”.

While polyethoxylated tallow amines (POEAs) were banned in Europe in 2016 due to their toxicity, they are among the multiple co-formulants authorized in GBHs in Canada. PMRA’s glyphosate’s re-evaluation in 2017 exceptionally assessed the toxicity of the surfactant POEA, only to conclude that: “No human health risks of concern were identified for these end-use products, provided that they contain no more than 20% POEA by weight” [45]. In contrast, one of the many studies on GBH formulations concluded that “the strong herbicidal and toxic properties of its formulations were exerted by the POEA formulant family alone. The toxic effects and endocrine disrupting properties of the formulations were mostly due to the formulants and not to G[lyphosphate]” [44]. Another study concluded that the extensively used GBH RangerPro containing POEA (POE-15) was far more cytotoxic than glyphosate [46]. Nevertheless, the PMRA ignores independent scientific literature on the harmful effects of GBH formulations.

### The Reauthorization of Glyphosate in Canada Based on Agrochemical Industry Studies

The evidence used by the PMRA to re-evaluate glyphosate raises serious questions about corporate influence. The PMRA based their re-evaluation on 32 pages of references to studies and information provided by the agrochemical industry, which were kept classified, and which were not subject to independent scientific peer review. In comparison, the review only made reference to about 15 pages of published studies and information, despite the voluminous published scientific literature on the subject [47]. The section concerning the PMRA’s evaluation of “toxicological hazards” was based on 118 references coming from the industry and seven “published” references which had no authors or places of publication identified [48].

These references did not correspond to the current state of scientific knowledge and were also seriously outdated: 81% were produced between 1972 and 1996. The majority of them date between 1987 and 1996, a period of sustained activity on the part of industry aimed at allowing the introduction of genetically-modified (GM) crops such as maize, soybeans, and canola in Canadian agriculture. It is doubtful whether these studies, conducted by pesticide producers prior to the marketing of GM crops that have caused GBH use to explode, are likely to shed light with any independence, credibility, or solid scientific assessments on the impact of these herbicides on human health [48].

A Pubmed (US National Library of Medicine) search of scientific articles on glyphosate done in 2017 identified 2400 published references since 1975, the majority of which (68%) were published from 2007 to 2017. The “toxicological hazards” section of the 2015 PMRA document, however, only included 12 references from the period 2007 to 2012. The 118 industrial references used by the PMRA corresponded to less than 5% of the 2400 scientific articles in Pubmed [48].

The PMRA wrote in its final decision that it considered additional published scientific literature, but it should be noted that this literature contains many ghostwritten scientific articles sponsored by the agrochemical industry as revealed by the Monsanto Papers [49]. Numerous studies demonstrating the harmful impacts of GBHs were also dismissed. For example, the WHO’s International Agency for Research on Cancer (IARC) concluded, after a thorough review of independent scientific studies in 2015 on both glyphosate and GBH, that they are carcinogenic and genotoxic, glyphosate being carcinogenic to animals and probably carcinogenic to humans. It also found an association between non-Hodgkin’s lymphoma and exposure to glyphosate [27]. The PMRA rhetoric for excluding this specific study is quite enlightening, with the Agency stating that:

“In total, the PMRA, in cooperation with the USEPA, assessed a much larger and more relevant body of scientific information than was considered by the IARC. Conversely, in its evaluation of the carcinogenic potential of glyphosate, the IARC considered only published sources of toxicology data, which included the scientific literature and certain documents published by regulatory agencies. The IARC did not directly consider, or did not consider at all, unpublished toxicology studies that were.”

[45] (p. 19)

In a nutshell, the IARC study was considered biased because it only analyzed independent scientific literature and excluded studies and data produced by the firms.

Thus, the PMRA evaluated the “toxicological hazards” of glyphosate on the basis of references coming from the industry and dating back more than 20, 30 or 40 years. Given the extensive scientific literature on glyphosate and GBHs and the rapid evolution of the research on the health impacts of chemical substances and chemical cocktails, it is difficult to claim that PMRA’s evaluation had scientific rigor [48]. Unsurprisingly, given how the re-evaluation was conducted, the PMRA concluded that glyphosate was not genotoxic, was unlikely to pose a cancer risk to humans and did not have endocrine disrupting properties because there was “no convincing evidence” of such properties [45].

Therefore, for Health Canada, products containing glyphosate present an “acceptable risk” defined as “if there is reasonable certainty that no harm to human health, future generations or the environment will result from exposure to or use of the product, taking into account its conditions or proposed conditions of registration.” [42]. This last point is critical as it allows the PMRA, despite strong evidence of the deleterious impacts of GBH, to transfer responsibility for safety via product labeling from the manufacturer and the government, onto the individual using GBH. Thus, the PMRA has re-approved glyphosate until 2032, stating that: “Products containing glyphosate are unlikely to affect your health when used according to label directions”. It reached the same conclusion for the environment, the occupational and residential risks and the dietary risks from food and water [45].

The case of the re-evaluation of GBH in Canada clearly illustrates the several flaws of such a regulatory approach. The Canadian health and regulatory agencies do not now, nor have they ever, take new discoveries of the health and environmental impacts of pesticides and their long-term toxicities in whole ecosystems into consideration. Despite the extensive scientific literature on the health impacts of pesticides and the mechanisms involved, the scientific assessment and regulations in Canada do not take into account carcinogenicity, genotoxicity, mutagenesis, neurotoxicity, reprotoxicity or endocrine disruptors of formulations or the cumulative and synergistic effects of pesticides.

As a result of this regulatory approach, in 2021, out of 531 “active” pesticide ingredients banned in 168 countries, including 464 banned or not approved in the European Union, only 32 were banned in Canada (18 are banned and 14 others cannot be imported to the country) [38]. According to Pesticides Action Network (PAN) International data, 146 pesticides are considered “Highly Hazardous” (HH) by the FAO/WHO Joint Meeting on Pesticide Management (JMPM); of these, only 25 are banned in Canada. Even Brazil, with 133 banned pesticides, has more banned pesticides than Canada [38].

## 3. Deceiving the Public through Clouded Transparency and Misleading Data on Pesticide Sales

Although PMRA’s evaluation of pesticides are mainly based on the manufacturer’s studies and only the ingredient they declared “active”, this regulatory body is constantly repeating that its pesticide evaluation and decision-making processes are science-based. The public must take their word for it, as transparency on these processes is sorely lacking. The “identity and concentration of formulants and contaminants” in a pesticide, other than those considered to be of health or environmental concern, are regarded as Confidential Business Information (CBI) which is unavailable to the public [42], and even basic information on pesticides, such as accurate sales numbers, are not publicly disclosed.

In Canada, agrochemical firms can decide which information is considered as Confidential Business Information (CBI) and therefore, what may not be disclosed publicly, even through the Access to Information Act. It is possible to ask to gain access to the “Reading Room” at PMRA’s National Head Office in Ottawa to look at scientific test data sent by manufacturers for pesticide registration or re-evaluation that are not considered CBI. However, to do so, a person must first send an “application form to identify the data to be inspected” and “an affidavit made under oath, or a statutory declaration under the Canada Evidence Act” stating the purpose of the visit [49]. Once the request is approved, an appointment scheduled, and the enquirer’s identity proven, entrance into the “Reading Room” is permitted with only a pencil and paper in hand, and only if the enquirer has previously signed an agreement to let Health Canada photocopy handwritten notes after exiting the room, as cell phones, laptops and digital cameras must be left outside the room to prevent copying data. The room is monitored during the visit, and contains only a computer with disabled ports and no access to the internet or internal networks. This is how the PMRA claims it “facilitates transparency” on pesticide evaluation.

**Figure 1:**
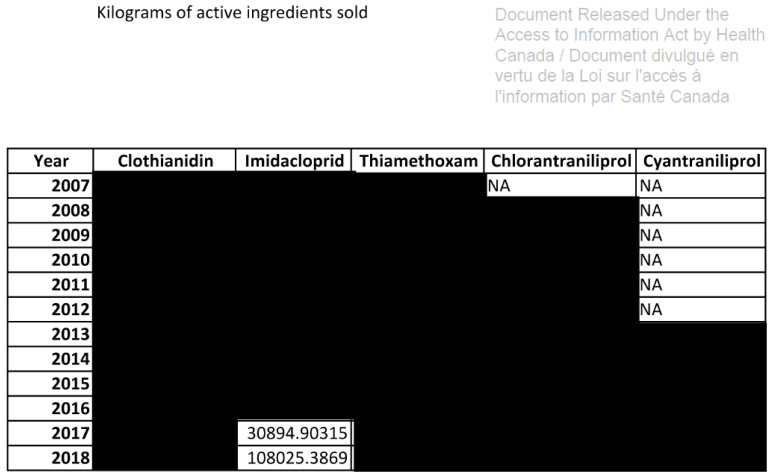
Canadian sales data of five neonicotinoids received from Health Canada following a request made under the Access to Information Act. The blackout data is censored, and NA means Not Available.

**Figure 2:**
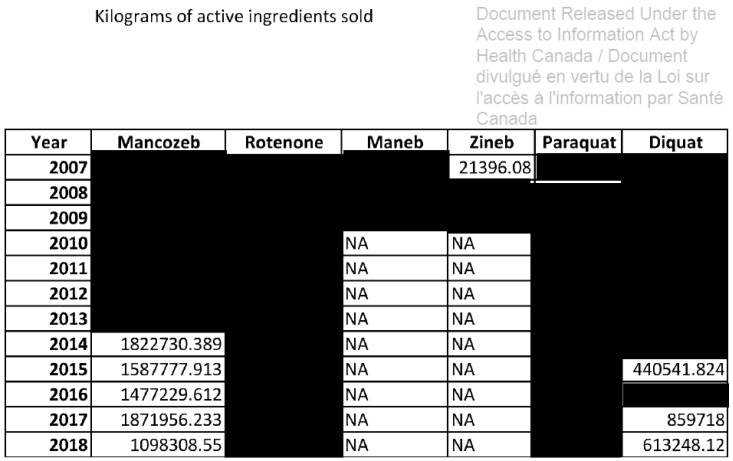
Canadian sales data of six pesticides linked to Parkinson’s disease received from Health Canada following a request made under the Access to Information Act. The blackout data is censored, and NA means Not Available.

The Canadian government does not even disclose specific pesticide sales data, publishing instead vague scales of quantities sold expressed with ˃ or < signs, for example: ˃ 1,000,000 kg of active ingredient (kg a.i.). This is misleading in terms of which products in what quantity are used in Canada and tends to underestimate sales. Following a request made under the Access to Information Act in 2021 to obtain the exact quantities of pesticides sold in Canada for several pesticides, the PMRA sent almost entirely blacked out data, notably for neonicotinoids (Figure 1), among which three were banned in EU in 2018, and many pesticides linked to Parkinson’s disease (Figure 2), as well as for atrazine, which has also been banned in Europe for 18 years, but not in Canada [25,50,51].

The scientific literature already shows that these pesticides are associated with Parkinson’s disease, a degenerative disease which heavily impacts both affected individuals and their relatives [52,53,54]. Syngenta, one of the main producers of the pesticide paraquat, is currently facing litigation brought by individuals with Parkinson’s disease in the United States. Syngenta has declassified internal documents in the course of this litigation, which confirm Syngenta’s long-standing knowledge of the harmful effects of paraquat [55].

Parkinson’s disease was recognized in 2021 as an occupational disease in the Canadian province of Quebec, and the first patient was compensated in 2022 [56,57]. Although paraquat, maneb, zineb and rotenone are not sold anymore in Quebec as of 2020, diquat and the fungicide mancozeb are still used, the latter even being the third most sold pesticide in that province with sales of 241,000 kg a.i. that year [58]. As this disease develops over years or even decades, historical data are of major importance. Given the wealth of evidence available regarding the known health risks of these pesticides, it is inadmissible and irresponsible that Health Canada refuses to publish data on these chemicals, which amounts to hiding important data from the public and researchers.

The data obtained for glyphosate, 2, 4-D and dicamba sales via the previously mentioned access-to-information request showed that between 2007 and 2018, total sales of “active” ingredients of these three major herbicides were in fact much higher than the suggested data in government’s sales reports (Table 1).

It is extremely concerning that Canadian authorities are hiding the precise sales data broken out by product names and co-formulants and contaminants in pesticide if their aim is, as claimed, to protect public health and the environment. Health Canada’s refusal to publicly disclose this information clearly hinders research on the harmful health and environmental impacts of pesticides.

### 3.1. Health Canada and Confidential Business Information (CBI)

It is important to note that this culture of secrecy with data and CBI is nothing new at Health Canada. The same issues existed for decades with pharmaceutical products, and Health Canada was considered proactive in restraining access to important data, such as adverse drug reactions (ADRs) [59]. Declared the most secretive of government departments by the Canadian Association of Journalists, Health Canada was awarded a “code of silence” award for its “remarkable zeal in suppressing information” and “concealing vital data about dangerous drugs” in 2004 [60]. Media pressure forced Health Canada to take steps toward greater transparency on drug information, and the information on ADRs was finally made publicly available online in 2005.

Health Canada has also faced serious criticism for its lack of transparency regarding clinical trial data considered CBI, which led to significant changes in 2014 after the passage of Bill C-17, an Act to amend the Food and Drugs Act—Protecting Canadians from Dangerous Drugs Act [61]. Under the transparency provisions of the law, companies must now make certain information about the clinical trials and other studies they sponsor in the course of developing a drug publicly available. In addition, the Minister for Health obtained new discretionary powers to share CBI without notice or consent from the party that claims ownership over that CBI. In particular, if the Minister believes there is a serious risk to human health, then CBI can be shared in an effort to avoid or mitigate that risk [62]. Even when no such risk appears to exist, the Minister still has the discretion to share CBI with anyone who “protects or promotes human health” or public safety, provided the person it is shared with does not use the CBI for commercial purposes [62].

These discretionary provisions gave the Minister the immediate ability to share unpublished safety and efficacy data related to therapeutic products—data which Health Canada had long refused to share on the grounds that it was CBI [63]. By incorporating the regulator’s understanding of what was CBI into the Food and Drugs Act, and by providing the regulator the capacity to determine what constitutes CBI under which circumstances, Bill C-17 opens up the possibility of disturbing Health Canada’s longstanding practices and lack of transparency by giving the federal Cabinet the power to pass new regulations specifying when certain information ceases to be CBI.

Considering that the Canadian health minister has acquired the capacity to define what constitutes CBI and what does not in order to better protect and promote public health, it is somewhat disconcerting that these powers are used only for improving the safety of therapeutic products, with the wall of silence remaining when it comes to agro-chemical products. It is particularly puzzling to observe this culture of silence from Health Canada’s PMRA when serious risks to human health have been confirmed in the scientific literature on pesticides.

### 3.2. Essential Information for Protecting Public Health and Ecosystems

The lack of accurate data on pesticide application also has impacts on our ability to assess their environmental damage, including to aquatic systems. In 2018 alone, according to the North American Pollutant Release and Transfer Register (PRTR), Canada and the United States released almost 379 million kilograms of pollutants that are developmental and reproductive toxins (on California Prop 65 List), known or suspected carcinogens, persistent, bioaccumulative and toxic, and metals into the Saint-Lawrence River watershed [64], a source of drinking water for millions of people.

As the Canadian National Pollutant Release Inventory (NPRI) includes limited pollutants from agricultural activities, the 324 million kilograms of pollutants declared on the Canadian side omits pesticides [64,65]. Yet, in Quebec, between 19 and 43 pesticides or pesticide degradation products were detected in rivers in agricultural regions pouring into the St. Lawrence River including the systematic detection of glyphosate and AMPA, atrazine, deethylatrazine (DEA), S-metolachlor, bentazon, chlorantraniliprole, imidacloprid, imazethapyr, and other worrying pesticides [66].

Whereas in many countries, such as France, the United States or Italy, there are databases and maps on the use, toxicity and presence of pesticides [67,68,69,70], in Canada and Quebec there are none, which hampers monitoring progress in reducing pesticide use or establishing correlations with health and environmental issues. According to data of exposure levels estimated by CAREX Canada (CARcinogen EXposure), back in 2016, the number of people living in areas with the highest potential for exposure to “glyphosate” was estimated to be 2,064,000 in Canada, of which more than half, 1,312,000 people, live in Quebec because an important part of the population reside near agricultural areas used for corn and soybean production [71]. As exposure to GBHs can lead to several diseases such as non-Hodgkin’s lymphoma (NHL) or other hematological malignancies, it is difficult not to be worried by the number of cases of NHL cancer which has increased in Canada (excluding Quebec) by 131% between 1992 and 2018 [72].

In Quebec, the number of NHL cases is even more alarming. In this province, herbicides represent 72% of pesticide sales in agriculture in 2019 [58] and, since 1992, GBH sales have been increasing every year and ended up being multiplied by 5.5 times. GBHs represented 54% of the sales in the agricultural sector in Quebec in 2019 [58] New NHL cases increased by 196% between 1984 and 2017 (58,481 cases in 2017) in Quebec but, in Montérégie and Estrie, two intensive agricultural regions where the population is the most exposed, a 247% and 335% increase respectively of new NHL cases was observed [73].

According to many studies, autism spectrum disorders (ASD) and learning disabilities in children are associated with pesticides [74,75,76] and recent research on “Endocrine and Nervous Disruptors”, or ENDs, shows these chemicals likely promote autism, depression, behavioral problems, diseases of nervous systems, degeneration, and more in humans [77]. In Quebec, between 2001–2002 and 2014–2015, there has been an 850% increase in the prevalence rate of children aged from 4 to 17 years with autism spectrum disorders (ASD) which represented 1 in 76 children [78]. A phenomenal increase was also observed in Montérégie, the region in Quebec with the most cases of children with ASD, with a prevalence of 1 in 49 children with ASD in 2014–2015, of which 83% were boys [79,80]. These statistics underline the urgent need for research on this issue.

Another matter is that the lack of data does not only concern pesticides, but diseases and health problems as well. Over a span of 10 years, from 2011 to 2021, Quebec had completely stopped publishing statistics on cancers in the province. Even though their new database is a significant improvement in terms of transparency and usability, their use of a different methodology, which is allegedly the reason for this 10 years gap, has made it impossible now to compare cancer rates in this province with the rest of Canada. There is also no database on diseases or other health problems (Parkinson’s, Alzheimer’s, ASD, learning disabilities, reproductive issues, miscarriage, low birth weight, etc.) and even less by specific geographic areas. The absence of such information and tools prevents researchers from conducting health studies and establishing correlations between the presence of and exposure to pesticides and diseases. Thus, collecting and disclosing data on pesticides as well as pollution and health problems are essential to protect human and ecosystem health.

## 4. Behind-the-Scenes Corporate Strategies Detrimental to Public Health

Monsanto has had, since its creation, a long history of using manipulation tactics to hide the toxicity of its products and their harmful impacts [81,82,83,84]. The Monsanto Papers, 10 million pages of internal documents from Bayer-Monsanto that were declassified during the first American trials lawsuits brought by 125,000 victims of non-Hodgkin’s lymphoma (NHL) attributed to Roundup use, have revealed surprising corporate maneuvers to conceal GBH toxicity, manipulate science and subvert public regulations intended to protect health and the environment: systematic ghostwriting of scientific articles; disrupting the peer-review process of scientific article publishing; creating false scientific and public controversies; personalized lobbying; orchestrating PR-disinformation campaigns; hiring regulatory and scientific consultancy and “product defense” firms (e.g., Intertek, Exponent, Gradient, etc.); intimidation, attack and destruction of the reputations and work of scientists, journalists and research centers; political and scientific lobbying organizations; keeping records on opinions and activities of thousands of persons; and influence peddling [85,86,87,88,89,90,91,92,93]. For example, molecular biologist Gilles-Éric Séralini who conducted several important scientific studies on GBH impacts and harms with his team, saw his name mentioned 55,952 times in the Monsanto Papers; the company clearly considered him to be an important target to be neutralized [91].

These strategies are not exclusive to the agrochemical sector, since they have also been used for several decades by firms in other industrial sectors such as pharmaceuticals [94,95,96], tobacco [97,98], asbestos [99], or food and alcohol [100,101]. In fact, when health risk assessment is central in determining the profitability of certain products, these behind-the-scenes corporate strategies deployed to influence science, regulation and public opinion in order to promote commercial imperatives over public health seem more like the norm than the exception [102,103,104]. Of particular importance is the capacity to influence and capture the scientific and regulatory processes. Regulatory capture can be defined as the “result or process by which regulation, in law or application, is consistently or repeatedly directed away from the public interest and toward the interests of the regulated industry, by the intent and action of the industry itself” [105]. If one agrees with this definition, then the Canadian PMRA can be considered a poster child of regulatory capture.

While it is not possible here to catalogue all of the problematic corporate strategies identified, we can identify three different types that have been central in the commercial success of GBHs in Canada: (1) the corporate shaping of the scientific narratives about the safety of glyphosate; (2) the hiding of risks associated with co-formulants in GBHs; and (3) the enduring intimacy between agrochemical companies and regulatory agencies.

### 4.1. Shaping the Scientific Narratives about the Safety of GBH

The shaping and monitoring of narratives and information on GBHs, and in particular of their health and environmental impacts, has been a priority from the start for Monsanto. By interfering with scientific assessment of pesticides, cultivating scientific doubt and confusion, as well as influencing and controlling the regulatory and political processes, Monsanto was working to keep what it called its “Freedom to Operate” [106]. Their greatest weapons have been the use of “ghost-management” [96,104], systematic hidden efforts and strategies routinely deployed by large corporations to shape social and informational structures in ways that benefit their commercial interests. These strategies have been shown in particular in the book by Seralini and Douzelet, The Monsanto Papers: Corruption of science and grievous harm to public health [107,108].

The case of Henry Miller, a prominent spokesperson in favor of GMOs and GBHs, who opposed the precautionary principle, is a good example of pro-active participation in shaping narratives and information. Before working with Monsanto, Miller was a U.S. Food and Drug Administration (FDA) employee and director of the FDA’s office of Biotechnology. He then worked to counter proposed EPA regulation he deemed “excessive” on GMOs and biotechnology, which he considered to be “environmentally friendly and pose negligible risk” that had “the potential to make many chemical pesticides obsolete” [109]. These are ideas that have long been conveyed by biotech firms. The Tobacco Papers revealed that, as early as 1998, Miller was promoting ideas, principles and concepts that the PMRA and other scientific and regulatory agencies worldwide would eventually adopt, and which have had important negative impacts on public health protection.

Among Miller’s principles, three have been central in shaping the frameworks to assess pesticides. First, the idea that it is hazardous to apply the “precautionary principle” to government oversight and the importance of using “comparative risk assessment”, which became central in the Canadian regulatory system. Second, the principle that “association is different from causality”, which prevented banning chemicals and pesticides for a very long time, even though correlations between health issues and pesticides had been established. Finally, the principle that “the dose makes the poison”, which is particularly inaccurate in the case of endocrine disrupting pesticides and synergistic effects of chemicals, was also a key element of Miller’s Work Plan Promoting Sound Science in Health, Environment, and Biotechnology Policy [109]. This plan, riddled with problematic claims and falsehoods, also underlined the importance of adopting “Sound Science” over “Junk Science”. According to Miller, government agencies were promoting self-interest, junk science, and hysteria by ignoring scientific principles and raising “false alarms over ‘grave dangers’ posed by dioxin, asbestos [...]”. Miller proposed a communication plan to counter “misinformation and disinformation” and promote “sound science and regulation”. Through his extensive writing in the press and his work at the Stanford Hoover Institute, sometimes directly ghostwritten by Monsanto [110], he was thus instrumental in propagating a narrative that served the commercial interests over the integrity of science and the imperatives of public health.

Shaping and monitoring the narratives surrounding GBHs was a constant priority for Monsanto in regulatory debates. For example, any threat to the global Roundup market had to be dealt with quickly, and Monsanto did not hesitate to intimidate and outright discredit researchers defending evidence-based narratives. When IARC announced its intention to evaluate glyphosate, it implied a large-scale evidence-based evaluation of glyphosate excluding biased corporate studies. In a message to another Monsanto scientist, Monsanto Company Lead Toxicologist, Donna Farmer wrote to John Acquavella: “Just wanted to let you know that what we have long been concerned about has happened. Glyphosate is on for an IARC review in March of 2015” [111]. Monsanto was also very worried that IARC’s conclusion would compromise the upcoming glyphosate re-evaluation by national regulatory agencies in Canada, USA and Europe. Thus, two months later Monsanto launched a $17 million USD disinformation campaign to discredit IARC’s work, assessment, and scientists [112]. Monsanto “vilified IARC’s work as “junk science”—a selective “cherry-picking” of data, based on an “agenda driven bias” [113].

Monsanto also mobilized people and organizations all over the world to undermine IARC, which was even accused of fraud. President and CEO of the American Chemistry Council, Cal Dooley, said in a press release: “There is an urgent need to fundamentally reform IARC’s Monographs program in order to stop the public confusion and hysteria around cancer prevention” [114]. Efforts to cut off IARC funding involved, among other things, Croplife International, a powerful worldwide lobbying organization, trying to influence Canadian representatives of IARC’s governing council in order to reduce Canadian funding of IARC [113]. As a result of this orchestrated campaign, “the credibility and integrity of IARC’s work are being challenged, its experts are being denigrated and harassed by lawyers, and its finances weakened” [113]. In the end, both the Canadian and American regulatory bodies dismissed the IARC conclusion in their glyphosate assessment.

### 4.2. Hiding Risks Associated to Surfactants: The Selective Production of Ignorance

A second fundamental strategy for Monsanto has been to hide the toxicity of GBH formulations that the research of Seralini and his team have demonstrated over the past 20 years [115]. The dissociation between the declared “active” ingredient (G) and the formulation (GBH) was essential to achieve this. Monsanto knew very well that the co-formulants that they had declared “inert” to regulatory authorities were in fact toxic.

Twenty years ago, in 2002, W. Heydens, Monsanto Company Product Safety Assessment Strategy Lead wrote, “formulated product (and thus the surfactant) does the damage” so “we are in pretty good shape with glyphosate but vulnerable with surfactants” [116]. One year later, Donna Farmer stated: “you cannot say that Roundup is not a carcinogen, we have not done the necessary testing on the formulation to make that statement.” [117]. But Monsanto internal emails showed that the Monsanto executive were in fact long aware of the glyphosate link to non-Hodgkin’s lymphoma [118] and that they had detailed “suspicions that formulated roundup can lead to tumor production” [119]. This knowledge of the harmful impact of GBH formulations prompted Monsanto Chemistry Regulatory Affairs Manager, Steven Adams, to write in 2014, “Don’t Want to Draw Attention to the Toxicity of Our Product” [120]; they tried to avoid any references to human health impacts. By focusing the regulatory debate over glyphosate instead of GBHs, Monsanto was able to downplay the harms and risks associated with its products [107].

Monsanto never hesitated to hide away the toxicity of GBH. In California, Proposition 65, also known as the Safe Drinking Water and Toxic Enforcement Act, requires businesses to provide warnings about significant exposures to chemicals that cause cancer, birth defects or other reproductive harm. California is thus publishing a list of chemicals, updated at least once a year, which now includes almost 1000 chemicals [121]. In order to avoid a Proposition 65 warning on Roundup, which would have affected its sales, Monsanto pressured Azko Nobel, one of two main manufacturers of C-6330 surfactant, to take off a Prop 65 cancer warning from their surfactant material safety data sheets [122]. The manufacturer agreed, which allowed Monsanto to delay the time before glyphosate would appear on the list by four years.

As early as 2010, Monsanto had developed its strategy to hide the toxicity of surfactants in general and POEA in particular at the international level [91]. When it was not possible to hide the toxicity of GBH surfactants, Monsanto tried instead to influence authorities regarding the language used in order to downplay health impacts. In France, a ban of Roundup POE-Tallowamine (POEA) surfactant came into effect in 2016. On the occasion of a planned visit to the French Embassy, David Carpintero, Former Monsanto Europe Corporate Affairs Lead for Crop Protection, tried to limit the damage of this ban by drafting clear guidelines to be communicated to French authorities:

“We simply would need the argumentation for the ban/withdrawal to not be based on ‘human health’ but other considerations like precautionary principle. The consequences of this ban if referring to human health risks have the potential to go beyond France and would potentially have global and trade impact. It is therefore of essence that any intention to ban does not refer to imminent human health risk.”

[123]

It is worth recalling that Canada re-approved POEA in GBHs the following year.

### 4.3. Enduring Intimacy between Agrochemical Companies and Regulatory Agencies

Canadian authorities have always been very attentive and responsive to biotech and pesticide companies’ narratives and demands. Canada was one of the first countries after the United States to approve GMOs, and still refuses mandatory GMO labeling. The PMRA, in particular, is still shrouded in a culture of secrecy, refusing to make important data about the health safety of GBHs publicly available. The intimacy between the public regulatory bodies and the agrochemical industry can hardly be overstated.

Regulatory capture results from the intensive lobbying of the Canadian federal government, which then embraces the narrative and arguments of the industry to the detriment of public health. For instance, Pierre Petelle, the President and CEO of Croplife Canada, had 61 registered meetings with key members of Parliament, governmental agencies directors, and political and ministerial advisors since May 2021 [124]. These meetings with mainly agents from the PMRA, Health Canada, Agriculture and Agri-Food Canada (AAFC) and the ministerial Standing Committee on Agriculture and Agri-Food which studies issues, bills and government activities related to Canada’s agriculture and agri-food industry, had, and still has, a major influence on the evaluation and regulation of pesticides in Canada.

The system of “revolving doors” between the private and public sectors, which refers to people moving from firms to strategic positions within public agencies, also plays an important role in shaping pesticide regulation, by positioning people who are particularly attentive to the demands of agrochemical companies or even acting in their interests within regulatory bodies. When the regulators and the regulatees share the same background, language, culture, and perceived interests, it creates an epistemic community where the only language spoken is the one shaped by the industry, which becomes a case of cultural capture [125]. Monsanto has been using this tactic of revolving doors extensively to get products approved and GMO labeling rejected [84,126].

Recent scandals involving Health Canada revealed this unhealthy intimacy between public regulatory bodies and agrochemical companies to the public. In 2021, the PMRA proposed, at the request of Bayer-Monsanto, to double or even quadruple the residue levels of glyphosate allowed in certain basic food products [127], causing a public outcry [128]. In order to understand the reason and assess the evidence supporting the decision, Vigilance OGM submitted an “Access to information” request to obtain the studies used to arrive at that decision. Health Canada and PMRA did not even send heavily redacted documents; all that arrived were 229 blank pages [129].

In 2022, Health Canada used files from the Croplife agrochemical lobby to announce that they would not evaluate nor regulate new gene-edited seeds, and that corporations would not even have to disclose to the government or the public that these seeds are gene-edited [130]. These gene drive organisms (GDO) enabled by new genetic engineering techniques such as CRISPR/Cas9, have a specifically altered genome that, in contrast to previous GMOs, are inherited by all offspring and thus spread genes synthesized in the laboratory into wild populations. Following this, 15 Canadian groups, including the National Farmers Union, sent a letter to Agriculture and Agri-Food Minister requesting that the president of the Canadian Food Inspection Agency (CFIA) be removed from his position: “The CFIA shows a long-standing pattern of deference to the regulated parties which has caused us to lose confidence in the CFIA’s ability to protect the public interest” [131].

## 5. Resorting to Legal Actions

Given the failure of public authorities to protect public health and the environment, or even respect their own legislative framework, there is an increasing resort to legal actions worldwide as well as in Canada [56]. There are now 500 registered legal cases linked to pesticides listed in the Justice Pesticides database, among which 160 are in the United States and 34 in Canada [132]. In a recent judgment, the Canadian Federal Court of Appeal, in a non-binding ruling, suggested the PMRA should reconsider appointing an independent scientific panel to review the safety and environmental assessment of glyphosate, re-approved in 2017, in response to the notice of objection filed by Safe Food Matters [133]. Moreover, this ruling asked the PMRA to clearly define “scientifically founded doubt” and “health risks” as well as to explain “the scientific approach it must take in evaluating the health and environmental risks”, thus underlining PMRA’s empty rhetoric.

Government decisions on “glyphosate” are also being legally challenged in the United States by non-governmental organizations (NGOs). In June 2022, a four-judge panel of the 9th United States Circuit Court of Appeals overturned the United States Environmental Protection Agency’s favorable human health safety assessment of glyphosate. The unanimous decision ruled that the EPA unlawfully approved glyphosate by ignoring rodent and epidemiological human studies that revealed harm, and by relying on studies that found no harm, and concluded that the “EPA’s errors in assessing human-health risk are serious” [134].

While these court rulings on the PMRA and EPA evaluation processes of glyphosate are certainly important, they received far less coverage than major trials, such as the ones against Monsanto. For the general public in North America, awareness as well as mobilization of civil society and researchers were largely linked to the high-profile trials that led to the publication of the Monsanto papers, which is in the process of being repeated with the publication of Syngenta’s internal documents showing its long-standing knowledge of the links between paraquat use and Parkinson’s disease.

## 6. Conclusions

Canada has a significant deficit in terms of responsible pesticide management compared to Europe and many other countries. At a time when it needs to significantly review and modify its regulatory and assessment frameworks for pesticides, the content of 2022 PMRA “public” consultations (by invitation only) on improving “transparency and stakeholder accessibility to information” and “modernizing business processes governing pesticide reviews” does not represent a significant reform. It does not propose to assess pesticide formulation instead of only the so-called active molecule, to base evaluation on recent independent scientific literature review considering genotoxicity, mutagenesis, and endocrine disruptors in pesticide formulation and pesticide cocktails, or even to give full public access to the data, studies used and evaluation process. Therefore, it falls short of the means that could protect public health and the environment.

Considering this last point, one might question why the regulation of pesticides is governed by the Ministry of Health’s Pest Control Products Act (PCPA) and not by the Canadian Environmental Protection Act (CEPA). CEPA, which was enacted in 1999, is currently being revised to recognize that every person in Canada has a right to a healthy environment and to consider, among other things, new knowledge about the toxicity of substances, including reproductive and endocrine toxicity, cocktail, and cumulative effects.

The agrochemical industry’s rhetoric and influence have seized Canadian public regulatory bodies to such an extent that even though an abundance of scientific literature demonstrates the health effects of many pesticides, Health Canada still delays withdrawing the most toxic ones from the market. Public authorities have not even elaborated on a plan to substantially reduce the use of major herbicides, and in particular GBHs, which are by far the most widely used pesticides in Canada. Considering that Canada is a major exporter of intensive crops (soybeans, canola, wheat, legumes, etc.) using a considerable amount of fertilizers and pesticides which often contain petroleum, as well as the world’s fourth largest oil producer, the importance of policies favoring the agrochemical industry is hardly surprising. Yet the socio-economic, health and environmental costs of pesticide use are much higher than the direct profit made by pesticide manufacturers, and these staggering costs in terms of environmental and health burden are assumed by citizens, communities and the public sector.

By embracing industry narratives and biased evidence, by being receptive to industry demands, and by opaque decision making that promotes commercial interests over the imperatives of health protection, PMRA’s pesticide assessments and regulations are thus consistently and repeatedly directed away from the goal of public health protection and toward the interests of the regulated industry.

The behind-the-scenes strategies deployed by the agrochemical industry have eroded research, regulation, public health, and the environment, as well as democratic processes, while generating immense profits for the companies. These industrial strategies and influences have percolated into governmental, scientific, public and information structure in such fundamental ways, that they prevent even the possibility of being aware of these manipulations and their implications. Resorting to legal actions, although an arduous and costly process, currently seems to be one of the only ways to expose these captures and challenge the present governmental framework and practices.

## Figures and Tables

**Table 1:** Precise Canadian total sales of glyphosate, 2,4-D and dicamba in kilograms of active ingredient (kg a.i.), from 2007 to 2018, obtained through a request made under the Access to Information Act compared to the declared total sales in government’s sales reports.

Name of “Active” Ingredient	Declared Total Sales	Precise Total Sales
**Glyphosate**	> 300,000,000 kg a.i.	469,839,370 kg a.i.
**2,4-D**	>11,000,000 kg a.i.	33,277,977 kg a.i.
**Dicamba**	>1,900,000 kg a.i.	5,102,149 kg a.i.

## Data Availability

Data sharing not applicable.
